# The Role of Glycocalyx Diversity and Thickness for Nanoparticle Internalization in M1-/M2-like Macrophages

**DOI:** 10.1021/acs.nanolett.4c04004

**Published:** 2024-12-02

**Authors:** Yu Liu, Yubei He, Han Xu, Amani Remmo, Frank Wiekhorst, Felix Heymann, Hanyang Liu, Eyk Schellenberger, Akvile Häckel, Ralf Hauptmann, Matthias Taupitz, Yu Shen, Emine Yaren Yilmaz, Dominik N. Müller, Luisa Heidemann, Robin Schmidt, Lynn Jeanette Savic

**Affiliations:** † Department of Radiology, Campus Virchow-Klinikum (CVK), Charité-Universitätsmedizin Berlin, Berlin 13353, Germany; ‡ Experimental and Clinical Research Center, a joint cooperation of Max Delbrück Center for Molecular Medicine and Charité-Universitätsmedizin Berlin, Berlin 13125, Germany; § Physikalisch-Technische Bundesanstalt, Berlin 10587, Germany; ∥ Department of Hepatology, Campus Virchow-Klinikum (CVK), Charité-Universitätsmedizin Berlin, Berlin 13353, Germany; ⊥ Department of Radiology, Campus Charité Mitte (CCM), Charité-Universitätsmedizin Berlin, Berlin 10117, Germany; # Department of Radiology, Campus Benjamin Franklin (CBF), Charité-Universitätsmedizin Berlin, Berlin 12203, Germany; ∇ Deutsches Rheuma-Forschungszentrum (DRFZ), Berlin 10117, Germany; ● Max Delbrück Center for Molecular Medicine in the Helmholtz Association, Berlin 13125, Germany; ○ Charité-Universitätsmedizin Berlin, Berlin 13125, Germany; ▲ Berlin Institute of Health at Charité-Universitätsmedizin Berlin, Berlin 10178, Germany

**Keywords:** M1/M2 macrophages, glycocalyx, nanoparticles uptake, VSOP, SPION

## Abstract

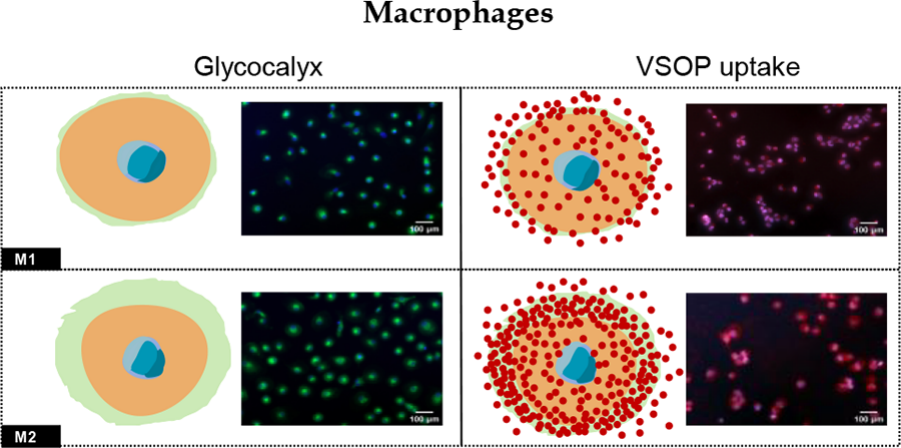

Very small superparamagnetic iron oxide nanoparticles (VSOPs) show diagnostic value in multiple diseases as a promising MRI contrast agent. Macrophages predominantly ingest VSOPs, but the mechanism remains unclear. This study identifies differences in VSOP uptake between pro-inflammatory M1 and anti-inflammatory M2 macrophages and explores the role of the pericellular glycocalyx. Glycosaminoglycans (GAG) synthesis activities and the pericellular glycocalyx for M1/M2-like macrophages were assessed by RT-qPCR, Click-iT reaction, and WGA-FITC staining. The uptake of europium-VSOP and Synomag by the two subtypes was measured using Prussian blue staining, fluorescent microscopy, and magnetic particle spectroscopy. The findings revealed that M2-like macrophages had higher GAG synthesis activity, a thicker glycocalyx, and increased nanoparticle uptake compared to M1-like macrophages. Enzymatic glycocalyx degradation significantly decreased nanoparticle uptake. This study demonstrates a positive correlation between glycocalyx and nanoparticle uptake that could be exploited for imaging and targeted therapy, particularly in cancer, where macrophage subtypes play distinct roles.

Superparamagnetic iron oxide nanoparticles (SPIONs) have emerged as a promising contrast agent in magnetic resonance imaging (MRI) owing to their unique properties, including superparamagnetism, biocompatibility, and tunable surface functionalities.^1^ Among SPIONs, very small superparamagnetic iron oxide nanoparticles (VSOPs), characterized by a diameter of approximately 7 nm and coated with biocompatible citric acid, revealed efficient cellular uptake and minimal cytotoxicity. Extensive preclinical and phase II clinical trials have underscored the versatility of VSOPs in various biomedical applications, including tumor and atherosclerotic plaque imaging, as well as mesenchymal stem cell tracking, and targeted drug delivery.^2^

Macrophages represent a pivotal role for VSOP internalization within the mononuclear phagocyte system since 95% of intravenously administered nanoparticles will be ingested by macrophages.^3^ In preclinical studies, SPIONs and particularly VSOPs were used to illustrate macrophages in the liver tumor microenvironment in a rabbit liver cancer model.^4−6^ The authors showed that peritumoral hypointensity in T2-weighted MRI sequences corresponded to iron deposition in macrophages surrounding the liver tumor.^7^ Macrophages play integral roles as immune regulators and effectors in diverse pathological contexts. Diverging into distinct phenotypic subsets, macrophages are broadly classified into M1 and M2 categories, each exerting unique immunomodulatory function. M1 macrophages, often referred to as classically activated macrophages, can exhibit potent antitumor capabilities through the secretion of cytotoxic molecules such as inflammatory cytokines.^8^ Conversely, M2 macrophages can promote tumor progression by fostering an immunosuppressive microenvironment and by promoting tumor cell proliferation, metastases, and angiogenesis.^9^ Particularly, tumor-associated macrophages (TAMs) are considered to exhibit M2-like, pro-tumorigenic traits associated with poor prognosis.^10^ Discerning the intricate balance between M1-like and M2-like macrophages holds profound implications for therapeutic interventions aimed at modulating the immune landscape within various disease settings.

The glycocalyx, a specialized structure situated on the extracellular surface of cell membranes, primarily comprises glycoproteins and glycosaminoglycans (GAGs). Its multifaceted functionality encompasses pivotal roles in cellular recognition, adhesion, regulation of permeability, and facilitation of phagocytic processes.^11^ Literature documents that VSOP can rapidly bind to the GAGs of macrophages’ glycocalyx due to their electrical charge, thereby initiating the critical priming step for the cellular uptake of VSOP.^12^ However, prevailing research efforts predominantly concentrate on elucidating the glycocalyx dynamics within vascular endothelial and epithelial cells, leaving a noticeable paucity in the exploration of pericellular glycocalyx properties in macrophages.

Therefore, this study aims to identify the differences in the uptake of VSOP between pro-inflammatory M1-like and anti-inflammatory, possibly protumorigenic M2-like macrophages, and to explore the potential impact of variations of the pericellular glycocalyx on VSOP uptake.

THP-1 cells were purchased from the American Type Culture Collection (ATCC) and cultured with complete RPMI medium (1640; Invitrogen, Germany). Primary PBMCs from a healthy donor were isolated following the instructions of BD Pharm Lyse Lysing Buffer (BD Biosciences, #555899). THP-1 cells and PBMCs were induced to polarize into M1-like and M2-like macrophages as the literature previously described. For details please refer to Supplementary Methods 1. The total RNA of cells was extracted with Trizol reagent (ABP Biosciences, #FP312A). The reverse transcription and real-time PCR were performed with the PCR kits (Invitrogen, #18064014) (Thermo Fisher, #A25776) according to the manufacturers’ instructions, respectively. For details please refer to Supplementary Methods 2 and *t*he sequences of the primers used are shown in Table 1 in the Supporting Information. For the GAG synthesis measurement, M1-like and M2-like macrophages in 8-well adherent chamber slides (Falcon, #354118) were incubated with RPMI medium containing Click-iT GalNAz (tetraacetylated N-azidoacetylgalactosamine, Invitrogen, #C33365) and Click-iT GlcNAz (tetraacetylated N-azidoacetylglucosamine, Invitrogen, #C33367) according to the manufacturer’s instruction. Then the washed cells were incubated with Alexa Fluor488 (Invitrogen, #C10405) and counterstained with Hoechst33342 (Thermo Fisher, #62249) for fluorescent microscopy. Further details can be found in Supplementary Methods 3. For the Glycocalyx immunohistochemistry staining, THP-1 cells were induced to polarized M1-like or M2-like macrophages in 8-well chamber slides. The cells were washed twice with PBS and fixed with cooled methanol and acetone 1:1 solution for 15 min at room temperature (RT). Afterward, the cells were washed twice with ddH2O and incubated with WGA-FITC (Gene-TEX, #GTX01502) at a concentration of 1:100 for 2 h at 37 °C. After washing again with ddH2O for 15 min, the cells were counterstained with Hoechst33342 at a concentration of 1:2000 for 15 min at RT. The staining was washed off again with ddH2O for 15 min in total, and the slides were sealed with mounting medium and observed with Axio Observer Z1 Zeiss microscope and analyzed by ImageJ as described above. Cell viability was evaluated using the 3-(4,5-dimethylthiazol-2-yl)-2,5-diphenyltetrazolium bromide (MTT) assay kit (Abcam, #ab211091) according to the manufacturer’s instructions. Further details can be found in Supplementary Methods 4. For Prussian blue staining of iron, cells were fixed in 4% formalin solution and then washed. Afterward, cells were incubated with potassium hexacyanoferrate (II) solution (Merk, #2440233) and HCl solution, then counterstained with nuclear red solution (Merck, #1001210500). Slides were washed and dehydrated for microscopy. For details please refer to Supplementary Methods 5. Regarding the fluorescent microscopy of europium to quantify the uptake of EU-VSOP into macrophages, cells were seeded into 8-well chamber slides and incubated with custom-made EU-VSOP.^13^ Then cells were washed before fixation with acetone-methanol (1:1). Afterward, the cells were counterstained with DAPI (Merck, #D9542–5MG) and washed with PBS. DELFIA Enhancer solutions (PerkinElmer, #C500–100) were added then fluorescent microscopy was performed. For details please refer to Supplementary Methods 6. Magnetic particle spectroscopy (MPS) was utilized to measure the dynamic ingestion of Synomag (micromod, #103–02–301) in macrophages. THP-1 cells were induced to polarized M1-like and M2-like macrophages and incubated with Synomag for 0h, 2, 4, 8, 12, 24, 48, and 60 h. At each time point, cells were collected and washed. Eventually, 10^6^ cells were diluted in 30 μL PBS and assembled in a 0.2 mL PCR tube (Thermo Fisher, #4316567). After placing the tube into the MPS pick-up coil, repetitive measurements were started without Synomag to check for magnetic impurities, before each sample was measured separately with MPS as previously described. For details please refer to Supplementary Methods 7. To digest the glycocalyx with hyaluronidase and heparinase III, adherent cells in chamber slides were washed three times with PBS, then incubated in RPMI without FBS containing hyaluronidase (Sigma, #H3506-100MG) and heparinase III (biotechne, #6145-GH) for 6 h with 5% CO_2_ at 37 °C. The digestion was terminated by the removal of the solution, and the cells were washed with PBS three times for subsequent glycocalyx staining with WGA-FITC. Additionally, the samples were measured with Prussian blue staining to determine the impact of the glycocalyx on the uptake of the nanoparticles. Statistical analyses were conducted using SPSS software (version 23.0) and GraphPad Prism (version 9.5.0). All data are presented as mean ± standard deviation (SD). For comparisons between two groups, the two-tailed student’s *t* test was performed. To determine the statistical differences of multiple groups, one-way repeated-measures ANOVA followed by the Bonferroni posthoc test was conducted. The experiments on THP-1 cells were repeated at least three times from independent samples. A p-value of less than 0.05 was considered statistically significant.

THP-1 treated with Phorbol 12-myristate 13-acetate (PMA) for 6 h and PBMC without any treatment were considered unpolarized macrophages (M0), respectively. Our results indicate that the M1 and M2 polarization of macrophages was induced successfully both in THP-1 and primary settings. Specifically, the normalized expression (compared with M0) of M1 polarization markers Tumor necrosis factor (TNF)-α and interleukin (IL)-1βwere increased in THP-1-derived M1-like macrophages compared with M2. The normalized expression of M2 polarization marker CD206 was increased in THP-1-derived M2-like macrophages. For macrophages derived from PBMCs, the normalized expression of TNF-αand IL-1βfor M1-like was increased compared with M2-like macrophages. The relative expression of CD206 in PBMC-originated M2-like macrophages was elevated compared with M1-like macrophages (Figure S1). All the above demonstrated that the M1-like and M2-like macrophages were induced successfully. To assess the effects of EU-VSOP and Synomag on macrophage proliferation, M1- and M2-like macrophages were differentiated from THP-1 cells and subsequently exposed to these nanoparticles. Cell viability remained largely unaffected over 72 h (Figure S2). Furthermore, to evaluate the impact of the nanoparticles on macrophages’ polarization, M0 macrophages derived from THP-1 cells were incubated with these nanoparticles, and the transcriptional markers of M1/M2 polarization were assessed. Results indicated that neither EU-VSOP nor Synomag induced significant polarization toward M1 or M2 phenotypes (Figure S2).

To verify the transcriptional expression of genes regulating the synthesis of GAGs, the genes involved in the synthesis of heparin sulfate (HS), hyaluronic acid (HA), and chondroitin sulfate (CS) were selected as these are the predominant components of GAGs in the glycocalyx. For THP-1-derived macrophages, the relative expression of exostosin glycosyltransferase (EXT)1, the key gene responsible for the synthesis of HS, was significantly increased in M2-like macrophages compared with M1-like macrophages (Figure 1a), Has1 and Has3 gene expression, the key genes for HA synthase, were also upregulated in M2-like macrophages in comparison to M1-like macrophages (Figure 1a). The expression of CHSY-1 encoding CS synthase was increased in M2-like macrophages as well (Figure 1a). Accordingly, the expression of ETX1, Has1, and Has3 was also increased in PBMC-derived M2-like macrophages (Figure 1b). Furthermore, we utilized metabolic assays to determine the GAG synthesis. After inducing the polarization of M1-like and M2-like macrophages, they were incubated with GalNAz and GlcNAz as part of the Click-iT assay, respectively. Consistent with prior observations, M2-like macrophages exhibited higher metabolism of GalNAz and GlcNAz compared to M1-like macrophages (Figure 1c and Figure 1d). The fluorescence intensity quantifying the utilization of GalNAz and GlcNAz in M1-like and M2-like macrophages were significantly distinct (Figure 1e and Figure 1f). These findings suggest that M2-like macrophages demonstrate a higher activity in synthesizing GAGs.

**Figure 1:**
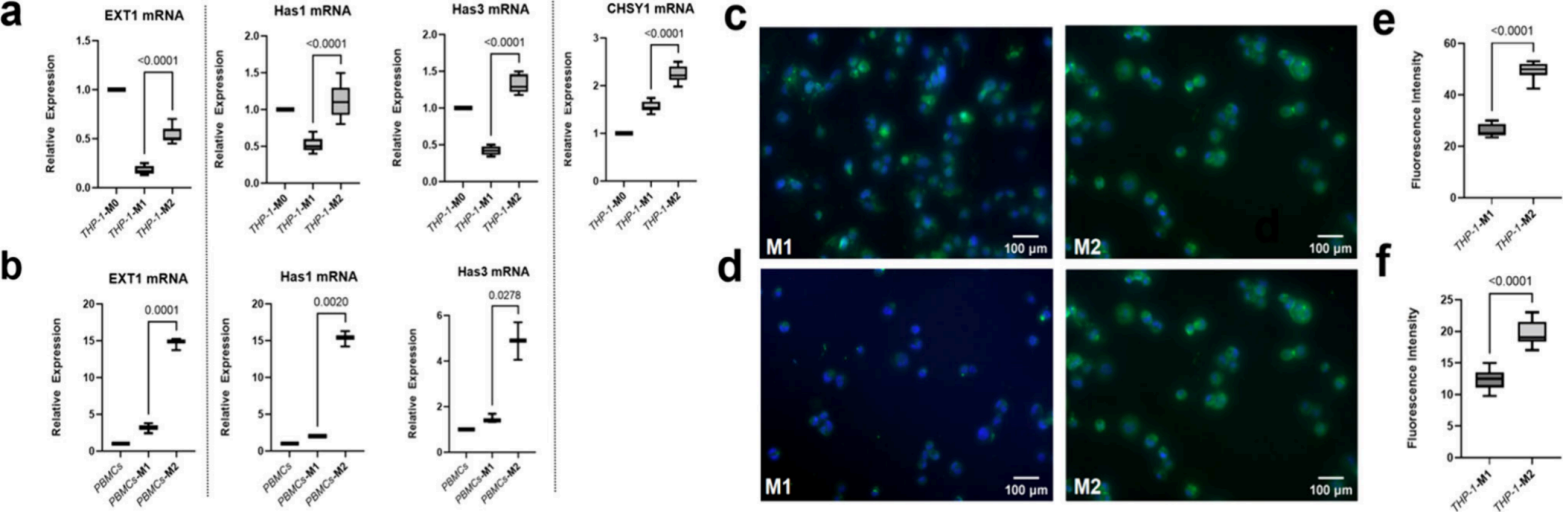
GAG synthesis activities were increased in M2-like macrophages compared with M1-like macrophages. The Whisker plots show the elevated expression of the GAGs synthesis regulatory genes in M2-like macrophages compared with M1-like macrophages derived from THP-1 cells (a) (*n* = 9) and PBMCs (b) (*n* = 3). Data were presented as mean ± SD. Two-tailed Student’s *t* test was performed for (a) and (b). Compared with M1-like macrophages derived from THP-1 cells, M2-like macrophages showed more potent uptake of metabolic substrates GalNAz (c) and GlcNAz (d). The green fluorescence intensity was quantified with ImageJ and made a comparison (e, f) (for (e) and (f), *n* = 9). Data were presented as mean ± SD. Two-tailed Student’s *t* test was performed for (e) and (f).

To visualize and compare the glycocalyx on M1-like and M2-like macrophages, we differentiated these macrophage types from THP-1 cells and PBMCs, followed by staining of the glycocalyx with WGA-FITC. The results showed that M2-like macrophages had a thicker glycocalyx layer than M1, both in THP-1-derived (Figure 2a and Figure 2b) and primary settings (Figure 2c and Figure 2d). The glycocalyx exhibits substantial variation in thickness and composition across different cell types. Its regulation by enzymes can modulate barrier and osmotic functions.^14^ While research on glycocalyx is more prevalent in vascular endothelial cells, studies investigating its presence on macrophages surfaces, particularly the disparities between M1-like and M2-like macrophages, remain scarce. Our findings represent, to our knowledge, the first report of significantly thicker glycocalyx on M2-like macrophages compared to M1-like macrophages. The thickness of the glycocalyx not only depends on the synthesis of GAGs but also on multifaceted regulatory processes influenced by several factors including cytokines. LPS, which was utilized to induce M1-like macrophages in this study, has been shown to activate heparinase, leading to glycocalyx degradation in macrophages.^15^ Additionally, IL-13 was reported to serve as a stimulant for the synthesis of HA and promote the HA deposition in the extracellular matrix. Moreover, the inhibition of the IL-13 signaling pathway has demonstrated a remarkable ability to boost HA synthase activity in murine models.^16^ Furthermore, IL-4 as used to induce M2-like macrophages, has been documented as a direct stimulator of GAGs production. Specifically, IL-4 exerts significant activation of metabolic pathways implicated in the generation of uridine diphosphate N-acetylglucosamine (UDP-GlcNAc) in macrophages, a crucial substrate involved in HA and HS synthesis.^17^

**Figure 2:**
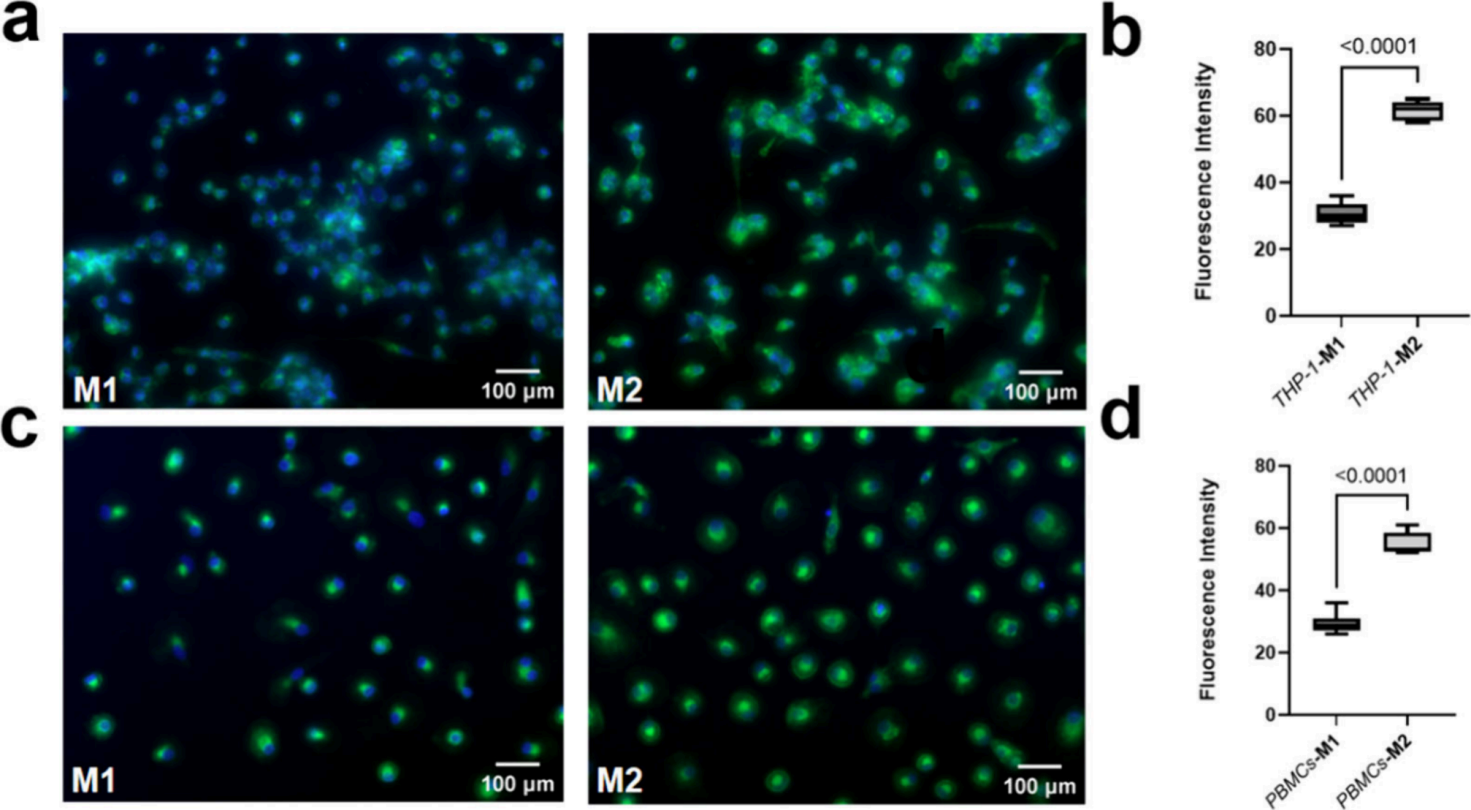
|The pericellular glycocalyx layer is thicker on M2-like compared with M1-like macrophages. M2-like macrophages reveal thicker pericellular glycocalyx compared with M1-like macrophages derived from THP-1 cells (a) and PBMCs (c). The green fluorescence intensity was quantified with ImageJ and made a comparison (b, d) (for (b), *n* = 9; for (d), *n* = 3). Data were presented as mean ± SD. Two-tailed Student’s *t* test was performed for (b) and (d).

To assess the uptake of VSOP by different macrophage subtypes, we utilized a derivative that contains Eu in the core of the VSOP, an element not naturally found in biological systems. EU-VSOP can be visualized using fluorescence microscopy following treatment with an enhancement solution, and quantified using fluorescence spectrophotometry.^18^ M1-like and M2-like macrophages derived from THP-1 cells and PBMCs were incubated with the same concentrations of EU-VSOP for 48 h. Subsequently, the internalized EU-VSOP was quantified by Prussian blue staining and fluorescence microscopy. The Prussian blue staining results showed that both THP-1 (Figure 3a and Figure 3b) and PMBCs-originated M2-like macrophages (Figure 3e and Figure 3f) ingested more EU-VSOP compared with M1-like macrophages. Additionally, fluorescent signal intensity was higher in THP-1-derived M2-like than M1-like macrophages (Figure 3c and Figure 3d). This is in contrast with earlier findings suggesting no significant differences between M1- and M2-like macrophages. However, discrepancies may arise from differences in cell models and polarization induction techniques.^19^ Variations in cell models and polarization induction methods may account for these discrepancies. Notably, other studies using the same cell models and techniques also support differential SPION uptake.^20^ Particularly, a bright circular signal was observed in M2-like macrophages but not in M1 (Figure 3g). The literature previously indicated rapid binding of VSOP and macrophage glycocalyx,^12^ specifically, Ludwig et al., demonstrated the interactions of the glycocalyx with nanoparticles using electron microscopy.^12^ The herein observed circular structure on fluorescence microscopy of Eu may resemble the EU-VSOP as they are internalized through the glycocalyx structures on the cell surface. Furthermore, results from our preliminary animal experiment revealed a spacial colocalization of M2-like macrophages, indicated by immunohistochemical staining for CD206, and Eu-VSOP deposition indicated by Prussian blue staining in the peritumoral zone on pathological sections of rabbit liver cancer tissues (Figure S4). Additionally, the uptake capacity of M1-like and M2-like macrophages was compared using another iron-containing nanoparticle that can be quantified using Magnetic Particle Spectroscopy (MPS).^21^ Synomag comprises 30-nanometer diameter multicore particles of maghemite crystals with surface-bound COOH groups, that facilitate an enhanced cellular uptake^22^ and superior MPS properties. First, Prussian blue staining confirmed increased iron density indicative of Synomag uptake into both THP-1 (Figure 4a and Figure 4b) and PBMC-originated M2-like (Figure 4c and Figure 4d) as compared to M1-like macrophages. Furthermore, dynamic MPS analysis at 0h, 2h, 4h, 8h, 12h, 24, 36, 48, and 60h revealed a faster and eventually increased Synomag ingestion in M2-like compared to M1-like macrophages (Figure 4e). VSOP demonstrate a broad spectrum of potential applications in clinical imaging with macrophages serving as primary targets. Given their pivotal role in atherosclerosis development, VSOP can effectively label extracellular components associated with plaque instability, aiding in the early identification of high-risk vulnerable plaques through imaging modalities.^12^ Furthermore, our findings suggest a novel avenue for VSOP utilization in tumor diagnosis and treatment. Immune evasion represents a critical hallmark of cancer including hepatocellular carcinoma (HCC), with M2-like macrophages in the tumor microenvironment being significant contributors to tumor invasion and metastases. Notably, immune cell infiltration, particularly by M2-like macrophages, escalates in HCC tissues following locoregional therapies such as transarterial chemoembolization (TACE) and ablation, correlating with a poorer prognosis.^23^ Thus, labeling M2-like macrophages with VSOPs in vivo may help characterize the tumor immune microenvironment and noninvasively detect possibly unfavorable macrophage subtypes on MRI that may require additional or more aggressive interventions in a personalized treatment fashion. Additionally, repolarizing M2 macrophages into antitumor M1 macrophages offers promise in enhancing macrophage-mediated tumor eradication.^9^ For instance, Egeblad et al. showcased its effectiveness in a mouse breast cancer model by employing a combination of IFN-γ with LPS/MPLA to induce M2-like macrophages conversion to the M1-like phenotype, resulting in substantial reductions in tumor burden.^24^ Nonetheless, the systemic administration of these drugs may cause undesirable systemic immune reactions. Our investigation underscores that M2-like macrophages exhibit higher VSOP uptake, offering a potential opportunity to leverage VSOP as a drug carrier for targeted delivery and M2 macrophages repolarization induction. Given that a significant portion of VSOPs is internalized by macrophages, particularly the M2-like subtype, this approach may be exploited as a nanodrug delivery system aimed at targeting and modulating M2 macrophages and enabling simultaneous diagnosis and treatment.

**Figure 3:**
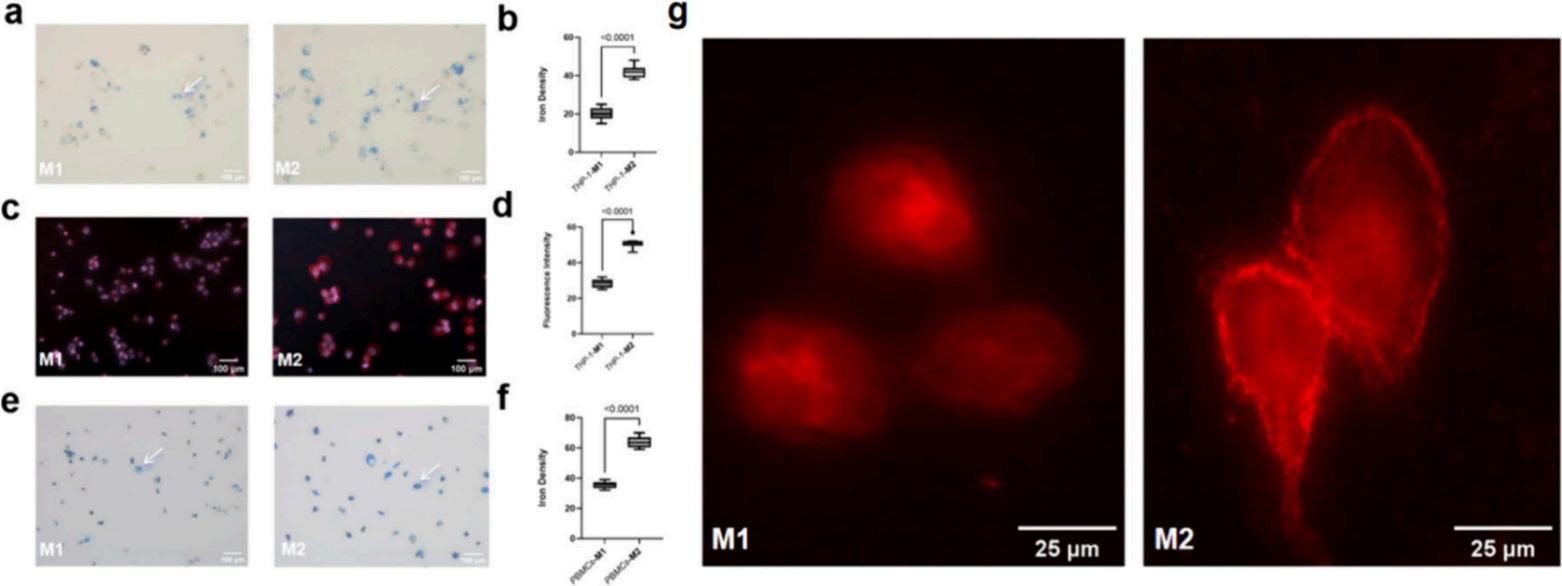
The EU-VSOP ingestion increased in M2-like macrophages compared with M1-like macrophages. M2-like macrophages ingested more EU-VSOP compared with M1-like macrophages derived with THP-1 cells (a) and PBMCs (e) validated by Prussian blue staining. It was confirmed with fluorescent microscopy for the macrophages derived from THP-1 cells (c). The iron density and red fluorescence intensity was quantified with ImageJ (b, d, and f) (for (b) and (d), *n* = 9; for (f), *n* = 3). The arrows point to the Prussian blue staining indicating iron content (a, e). The bright circular structure around M2-like macrophages is assumed to be glycocalyx with aggregated EU-VSOP, this structure did not appear on M1-like macrophages (**g**). Data were presented as mean ± SD. Two-tailed Student’s *t* test was performed for (b), (d), and (f).

**Figure 4:**
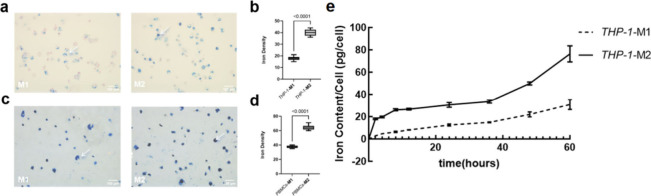
The Synomag internalization increased in M2-like macrophages compared with M1-like macrophages. M2-like macrophages ingested more Synomag compared with M1-like macrophages derived with THP-1 cells (a) and PBMCs (c) validated by Prussian blue staining. The arrows point to the Prussian blue staining indicating iron content (a, c). The iron density was quantified with ImageJ (b, d). Increased and faster uptake was observed in M2-like macrophages compared with M1-like macrophages derived from THP-1 cells through the dynamic uptake determination with MPS (e). (for (b), *n* = 9; for (d) and (e), *n* = 3). Data were presented as mean ± SD. Two-tailed Student’s *t* test was performed for (b), (d), and (e).

To further unravel the potential correlation between glycocalyx and nanoparticle ingestion, THP-1-originated M2-like macrophages were incubated with heparinase III and hyaluronidase simultaneously, which efficiently degrade HA and HS, the predominant components of the glycocalyx.^25^ A viability assay was conducted to confirm no relevant impact on cell viability of the enzymes at the working concentration and even twice the working concentration (Figure S3). WGA-FITC staining confirmed a significant reduction of the pericellular glycocalyx layer of M2-like macrophages (Figure 5a and Figure 5b). Additionally, a distinct reduction of EU-VSOP (Figure 5c and Figure 5d) and Synomag (Figure 5e and Figure 5f) ingestion in enzyme-digested M2-like macrophages was observed by Prussian blue staining which validated the positive association of the thickness of glycocalyx and nanoparticle uptake in macrophages. Furthermore, we repeated the experiment using macrophages derived from PBMCs, which demonstrated a consistent trend (Figure 5g, 5i, 5k, 5h, 5j, and 5l). This finding aligns with existing literature suggesting that the inhibition of GAG synthesis via glucose deprivation can notably diminish VSOP uptake by macrophages.^26^ However, the affinity of certain GAGs also impacts the binding and internalization of iron-containing nanoparticles by the glycocalyx. For example, HS forms strong coordination bonds with iron ions due to its sulfuric acid groups, enhancing its binding capacity with such nanoparticles. Additionally, HS chains are typically longer than HA, providing more binding sites and increasing binding efficacy.^27^ Additionally, IL-13 promotes changes in sulfation patterns within GAGs, which may also result in an increased uptake of iron nanoparticles.^28^ Moreover, artificial aggregation of gold nanoparticles enhances phagocytosis, potentially leading to high gold loading in cells.^29^ Similarly, VSOP accumulation on cell surfaces facilitates phagocytosis, suggesting that the glycocalyx of M2-like macrophages, rich in sulfated GAGs that bind VSOPs, may in turn also promote the ingestion of VSOPs.^12^

**Figure 5:**
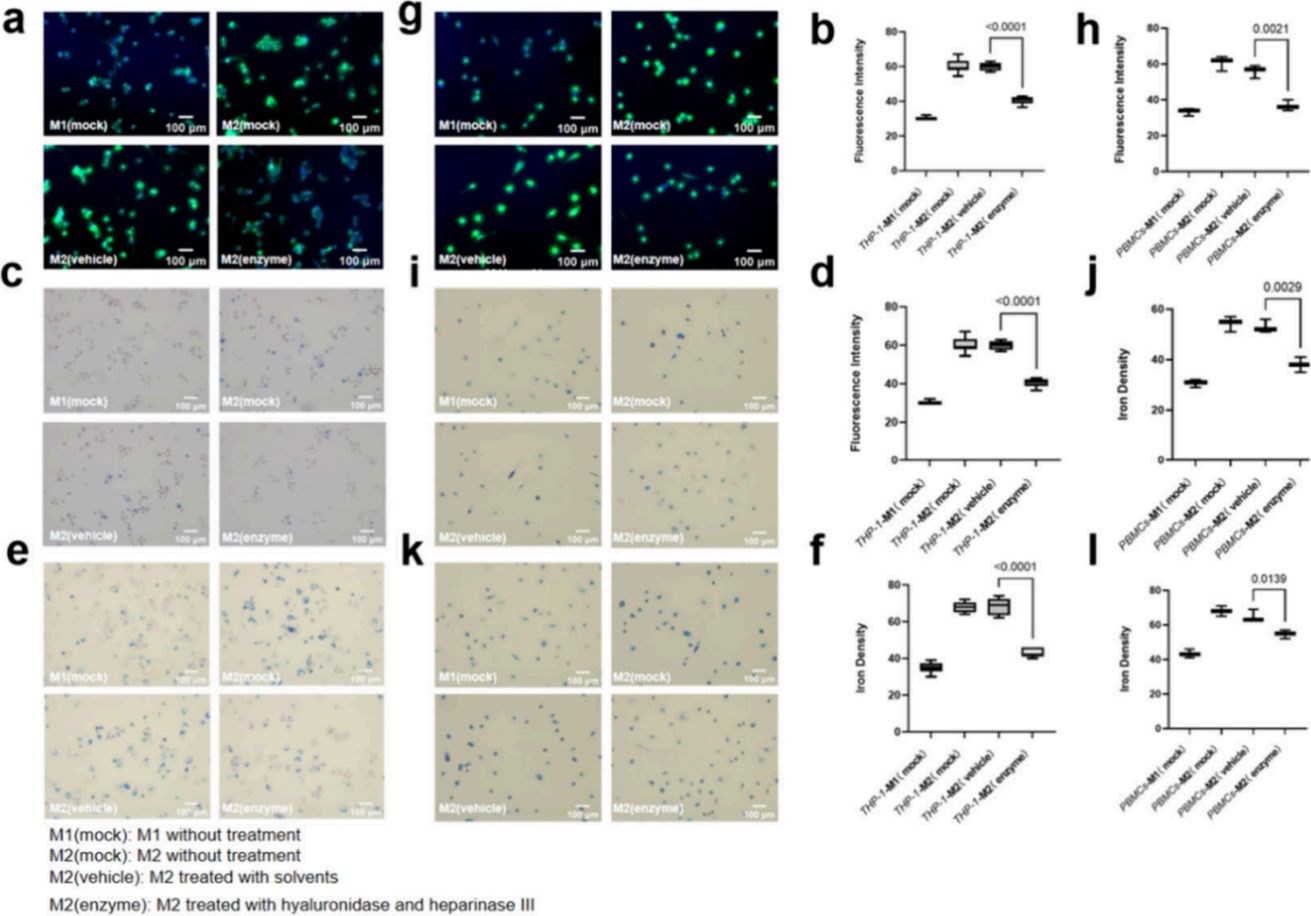
Digestion of glycocalyx with hyaluronidase and heparinase III reduced the nanoparticle internalization in M2-like macrophages. M1 and M2 derived from THP-1 cells (a) and PBMCs (g) were treated with hyaluronidase and heparinase III and then were stained with WGA-FITC and Hoechst33254, and the green fluorescence intensity was quantified with ImageJ (b, h). M1 and M2 derived from THP-1 cells and PBMCs were treated with hyaluronidase and heparinase III and then incubated with EU-VSOP for 48 h and the iron content was measured with Prussian blue staining (c, (i), then the iron density was quantified with ImageJ and made a comparison (d, j). M1 and M2 derived from THP-1 cells and PBMCs were treated with hyaluronidase and heparinase III and then incubated with Synomag for 48 h and the iron content was measured with Prussian blue staining (e, k), then the iron density was quantified with ImageJ and made a comparison (f, l). (for b, d and f, *n* = 9; for h, j and l, *n* = 3). Data were presented as mean ± SD. Two-tailed Student’s *t* test was performed for b, d, f, h, j and l.

This study reveals two main findings. First, marked differences in the glycocalyx composition were observed between M1-like and M2-like macrophage subtypes, with M2-like macrophages exhibiting upregulated GAG synthesis, resulting in a significantly thicker overall glycocalyx compared to M1-like macrophages. Second, we found that M2-like macrophages exhibit enhanced uptake of VSOP compared to M1-like macrophages, which is reduced after the degradation of GAGs on the surface of M2-like macrophages. This finding suggests a positive correlation between pericellular glycocalyx and nanoparticle uptake in macrophages, which could be exploited for contrast-enhanced imaging and potentially targeted therapy in cancer patients.

## Supporting Information Available

Sequences of the primers for Q-PCR, confirmation of polarization in M1-like and M2-like macrophages using real-time qPCR, effect of EU-VSOP and Synomag on the proliferation and polarization of macrophages, effect of hyaluronidase and heparinase III on the viability of macrophages, colocalization of CD206-positive macrophages and iron-deposition from EU-VSOP in the peritumoral zone, and additional experimental details, materials, and methods (PDF)

## Abbreviations

ATCC: American type culture collection
CS: chondroitin sulfate
EU-VSOP: europium-VSOP
GlcNAz: tetraacetylated N-azidoacetylglucosamine
GAG: glycosaminoglycan
GalNAz: tetraacetylated N-azidoacetylgalactosamine
HA: hyaluronic acid
HS: heparin sulfate
IFN: interferon
IL: interleukin
LPS: lipopolysaccharide
MPS: magnetic particle spectroscopy
MRI: magnetic resonance imaging
MTT: 3-(4,5-dimethylthiazol-2-yl)-2,5-diphenyltetrazolium bromide
PMA: phorbol 12-myristate 13-acetate
RT: room temperature
SD: standard deviation
SPIONs: superparamagnetic iron oxide nanoparticles
TAM: tumor-associated macrophage
TACE: transarterial chemoembolization
UDP-GlcNAc: uridine diphosphate N-acetylglucosamine
VSOPs: very small superparamagnetic iron oxide nanoparticles

## References

[ref1] Wei H.; Hu Y.; Wang J.; Gao X.; Qian X.; Tang M. Superparamagnetic Iron Oxide Nanoparticles: Cytotoxicity, Metabolism, and Cellular Behavior in Biomedicine Applications. Int. J. Nanomedicine 2021, 16, 6097–6113. 10.2147/IJN.S321984. 34511908 PMC8418330

[ref2] Zhou Y.; Dai Z. New Strategies in the Design of Nanomedicines to Oppose Uptake by the Mononuclear Phagocyte System and Enhance Cancer Therapeutic Efficacy. Chem. - Asian J. 2018, 13 (22), 3333–3340. 10.1002/asia.201800149. 29441706

[ref3] Cheng M. J.; Mitra R.; Okorafor C. C.; Nersesyan A. A.; Harding I. C.; Bal N. N.; Kumar R.; Jo H.; Sridhar S.; Ebong E. E. Targeted Intravenous Nanoparticle Delivery: Role of Flow and Endothelial Glycocalyx Integrity. Ann. Biomed. Eng. 2020, 48 (7), 1941–1954. 10.1007/s10439-020-02474-4. 32072383 PMC8025840

[ref4] Savic L. J.; Schobert I. T.; Peters D.; Walsh J. J.; Laage-Gaupp F. M.; Hamm C. A.; Tritz N.; Doemel L. A.; Lin M.; Sinusas A.; Schlachter T.; Duncan J. S.; Hyder F.; Coman D.; Chapiro J. Molecular Imaging of Extracellular Tumor pH to Reveal Effects of Locoregional Therapy on Liver Cancer Microenvironment. Clin. Cancer Res. 2020, 26 (2), 428–438. 10.1158/1078-0432.CCR-19-1702. 31582517 PMC7244230

[ref5] Savic L. J.; Doemel L. A.; Schobert I. T.; Montgomery R. R.; Joshi N.; Walsh J. J.; Santana J.; Pekurovsky V.; Zhang X.; Lin M.; Adam L.; Boustani A.; Duncan J.; Leng L.; Bucala R. J.; Goldberg S. N.; Hyder F.; Coman D.; Chapiro J. Molecular MRI of the Immuno-Metabolic Interplay in a Rabbit Liver Tumor Model: A Biomarker for Resistance Mechanisms in Tumor-Targeted Therapy?. Radiology 2020, 296 (3), 575–583. 10.1148/radiol.2020200373. 32633675 PMC7434651

[ref6] Van Breugel J. M. M.; Geschwind J.-F.; Mirpour S.; Savic L. J.; Zhang X.; Duran R.; Lin M.; Miszczuk M.; Liapi E.; Chapiro J. Theranostic Application of Lipiodol for Transarterial Chemoembolization in a VX2 Rabbit Liver Tumor Model. Theranostics 2019, 9 (13), 3674–3686. 10.7150/thno.32943. 31281506 PMC6587357

[ref7] Collettini F.; Brangsch J.; Reimann C.; Chapiro J.; Savic L. J.; Buchholz R.; Keller S.; Hamm B.; Goldberg S. N.; Makowski M. R. Hepatic Radiofrequency Ablation: Monitoring of Ablation-Induced Macrophage Recruitment in the Periablational Rim Using SPION-Enhanced Macrophage-Specific Magnetic Resonance Imaging. Invest. Radiol. 2021, 56 (9), 591–598. 10.1097/RLI.0000000000000777. 33787536

[ref8] Orecchioni M.; Ghosheh Y.; Pramod A. B.; Ley K. Macrophage Polarization: Different Gene Signatures in M1(LPS+) vs. Classically and M2(LPS-) vs. Alternatively Activated Macrophages. Front. Immunol. 2019, 10, 1084 10.3389/fimmu.2019.01084. 31178859 PMC6543837

[ref9] Wu K.; Lin K.; Li X.; Yuan X.; Xu P.; Ni P.; Xu D. Redefining Tumor-Associated Macrophage Subpopulations and Functions in the Tumor Microenvironment. Front. Immunol. 2020, 11, 1731 10.3389/fimmu.2020.01731. 32849616 PMC7417513

[ref10] Tiainen S.; Masarwah A.; Oikari S.; Rilla K.; Hämäläinen K.; Sudah M.; Sutela A.; Vanninen R.; Ikonen J.; Tammi R.; Tammi M.; Auvinen P. Tumor Microenvironment and Breast Cancer Survival: Combined Effects of Breast Fat, M2Macrophages and Hyaluronan Create a Dismal Prognosis. Breast Cancer Res. Treat. 2020, 179 (3), 565–575. 10.1007/s10549-019-05491-7. 31720917 PMC6997252

[ref11] Reitsma S.; Slaaf D. W.; Vink H.; Van Zandvoort M. A. M. J.; Oude Egbrink M. G. A. The Endothelial Glycocalyx: Composition, Functions, and Visualization. Pflüg. Arch. - Eur. J. Physiol. 2007, 454 (3), 345–359. 10.1007/s00424-007-0212-8. PMC1915585 17256154

[ref12] Ludwig A.; Poller W. C.; Westphal K.; Minkwitz S.; Lättig-Tünnemann G.; Metzkow S.; Stangl K.; Baumann G.; Taupitz M.; Wagner S.; Schnorr J.; Stangl V. Rapid Binding of Electrostatically Stabilized Iron Oxide Nanoparticles to THP-1 Monocytic Cells via Interaction with Glycosaminoglycans. Basic Res. Cardiol. 2013, 108 (2), 328 10.1007/s00395-013-0328-2. 23314954

[ref13] De Schellenberger A. A.; Hauptmann R.; Millward J. M.; Schellenberger E.; Kobayashi Y.; Taupitz M.; Infante-Duarte C.; Schnorr J.; Wagner S. Synthesis of Europium-Doped VSOP, Customized Enhancer Solution and Improved Microscopy Fluorescence Methodology for Unambiguous Histological Detection. J. Nanobiotechnology 2017, 15 (1), 71 10.1186/s12951-017-0301-6. 29017510 PMC5634840

[ref14] Lalisang T. J. M. dr. Biology of Glycocalyx: The Essential Role in Maintaining Epithelial Barrier: A Mini-Review. New Ropanasuri J. Surg. 2019, 4, 7 10.7454/nrjs.v4i2.1059.

[ref15] Tang Y.; Wang X.; Li Z.; He Z.; Yang X.; Cheng X.; Peng Y.; Xue Q.; Bai Y.; Zhang R.; Zhao K.; Liang F.; Xiao X.; Andersson U.; Wang H.; Billiar T. R.; Lu B. Heparin Prevents Caspase-11-Dependent Septic Lethality Independent of Anticoagulant Properties. Immunity 2021, 54 (3), 454–467. 10.1016/j.immuni.2021.01.007. 33561388

[ref16] Donlan A. N.; Sutherland T. E.; Marie C.; Preissner S.; Bradley B. T.; Carpenter R. M.; Sturek J. M.; Ma J. Z.; Moreau G. B.; Donowitz J. R.; Buck G. A.; Serrano M. G.; Burgess S. L.; Abhyankar M. M.; Mura C.; Bourne P. E.; Preissner R.; Young M. K.; Lyons G. R.; Loomba J. J.; Ratcliffe S. J.; Poulter M. D.; Mathers A. J.; Day A. J.; Mann B. J.; Allen J. E.; Jr W. A. P. IL-13 Is a Driver of COVID-19 Severity. JCI Insight 2021, 6, e150107 10.1172/jci.insight.150107. 34185704 PMC8410056

[ref17] Jha A. K.; Huang S. C.-C.; Sergushichev A.; Lampropoulou V.; Ivanova Y.; Loginicheva E.; Chmielewski K.; Stewart K. M.; Ashall J.; Everts B.; Pearce E. J.; Driggers E. M.; Artyomov M. N. Network Integration of Parallel Metabolic and Transcriptional Data Reveals Metabolic Modules That Regulate Macrophage Polarization. Immunity 2015, 42 (3), 419–430. 10.1016/j.immuni.2015.02.005. 25786174

[ref18] Millward J. M.; Ariza De Schellenberger A.; Berndt D.; Hanke-Vela L.; Schellenberger E.; Waiczies S.; Taupitz M.; Kobayashi Y.; Wagner S.; Infante-Duarte C. Application of Europium-Doped Very Small Iron Oxide Nanoparticles to Visualize Neuroinflammation with MRI and Fluorescence Microscopy. Neuroscience 2019, 403, 136–144. 10.1016/j.neuroscience.2017.12.014. 29273325

[ref19] Poller W. C.; Pieber M.; Boehm-Sturm P.; Ramberger E.; Karampelas V.; Möller K.; Schleicher M.; Wiekhorst F.; Löwa N.; Wagner S.; Schnorr J.; Taupitz M.; Stangl K.; Stangl V.; Ludwig A. Very Small Superparamagnetic Iron Oxide Nanoparticles: Long-Term Fate and Metabolic Processing in Atherosclerotic Mice. Nanomedicine Nanotechnol. Biol. Med. 2018, 14 (8), 2575–2586. 10.1016/j.nano.2018.07.013. 30179669

[ref20] Satomi T.; Ogawa M.; Mori I.; Ishino S.; Kubo K.; Magata Y.; Nishimoto T. Comparison of Contrast Agents for Atherosclerosis Imaging Using Cultured Macrophages: FDG Versus Ultrasmall Superparamagnetic Iron Oxide. J. Nucl. Med. 2013, 54 (6), 999–1004. 10.2967/jnumed.112.110551. 23670898

[ref21] Paysen H.; Loewa N.; Stach A.; Wells J.; Kosch O.; Twamley S.; Makowski M. R.; Schaeffter T.; Ludwig A.; Wiekhorst F. Cellular Uptake of Magnetic Nanoparticles Imaged and Quantified by Magnetic Particle Imaging. Sci. Rep. 2020, 10 (1), 1922 10.1038/s41598-020-58853-3. 32024926 PMC7002802

[ref22] Potlog T.; Popusoi A.; Lungu I.; Robu S.; Bulimestru I. Photophysics of Tetracarboxy-Zinc Phthalocyanine Photosensitizers. RSC Adv. 2022, 12 (49), 31778–31785. 10.1039/D2RA05676C. 36380933 PMC9639198

[ref23] Song H.; Wang X.; Zhang C.; He J. Construction of an M2Macrophage-Related Prognostic Model in Hepatocellular Carcinoma. Front. Oncol. 2023, 13, 1170775 10.3389/fonc.2023.1170775. 37409259 PMC10319018

[ref24] Sun L.; Kees T.; Almeida A. S.; Liu B.; He X.-Y.; Ng D.; Han X.; Spector D. L.; McNeish I. A.; Gimotty P.; Adams S.; Egeblad M. Activating a Collaborative Innate-Adaptive Immune Response to Control Metastasis. Cancer Cell 2021, 39 (10), 1361–1374. 10.1016/j.ccell.2021.08.005. 34478639 PMC8981964

[ref25] Kaur G.; Leskova W.; Harris N. R. The Endothelial Glycocalyx and Retinal Hemodynamics. Pathophysiology 2022, 29 (4), 663–677. 10.3390/pathophysiology29040052. 36548208 PMC9785437

[ref26] Cechowska-Pasko M.; Bańkowski E. Glucose Deficiency Inhibits Glycosaminoglycans Synthesis in Fibroblast Cultures. Biochimie 2010, 92 (7), 806–813. 10.1016/j.biochi.2010.02.029. 20219625

[ref27] Freise C.; Zappe A.; Löwa N.; Schnorr J.; Pagel K.; Wiekhorst F.; Taupitz M. Uremic Toxin-Induced Exosome-like Extracellular Vesicles Contain Enhanced Levels of Sulfated Glycosaminoglycans Which Facilitate the Interaction with Very Small Superparamagnetic Iron Oxide Particles. Int. J. Mol. Sci. 2023, 24 (18), 14253 10.3390/ijms241814253. 37762555 PMC10532171

[ref28] Allen J. E. IL-4 and IL-13: Regulators and Effectors of Wound Repair. Annu. Rev. Immunol. 2023, 41 (1), 229–254. 10.1146/annurev-immunol-101921-041206. 36737597

[ref29] Krpetić Ž.; Nativo P.; Prior I. A.; Brust M. Acrylate-Facilitated Cellular Uptake of Gold Nanoparticles. Small 2011, 7 (14), 1982–1986. 10.1002/smll.201100462. 21648075

