# Supplementary material for: The Role of Glycocalyx Diversity and Thickness for Nanoparticle Internalization in M1-/M2-like Macrophages
Source: Nano Lett. 2024 Dec 2;24(49):15607–14. doi: 10.1021/acs.nanolett.4c04004 (PMC11638944; doi:10.1021/acs.nanolett.4c04004)
Supplement: Supplementary file 1 — nl4c04004_si_001.pdf [file nl4c04004_si_001.pdf]

Supporting Information

The Role of Glycocalyx Diversity and Thickness for Nanoparticle Internalization in M1-/M2-like Macrophages

Yu Liu[1,2], Yubei He[1,2], Han Xu[1,2], Amani Remmo[3], Frank Wiekhorst[3], Felix Heymann[4], Hanyang Liu[4], Eyk Schellenberger[5], Akvile Häckel[5], Ralf Hauptmann[5], Matthias Taupitz[6], Yu Shen[7], Emine Yaren Yilmaz[1,2], Dominik N. Müller[2,8,9], Luisa Heidemann[1,2], Robin Schmidt[1,2], Lynn Jeanette Savic[1,2,10*]

1. Department of Radiology, Campus Virchow-Klinikum (CVK), Charité-Universitätsmedizin Berlin, Berlin, 13353, Germany
2. Experimental and Clinical Research Center, a joint cooperation of Max Delbrück Center for Molecular Medicine and Charité-Universitätsmedizin Berlin, Berlin, 13125, Germany
3. Physikalisch-Technische Bundesanstalt, Berlin, 10587, Germany
4. Department of Hepatology, Campus Virchow-Klinikum (CVK), Charité -Universitätsmedizin Berlin, Berlin, 13353, Germany
5. Department of Radiology, Campus Charité Mitte (CCM), Charité-Universitätsmedizin Berlin, Berlin, 10117, Germany
6. Department of Radiology, Campus Benjamin Franklin (CBF), Charité-Universitätsmedizin Berlin, Berlin, 12203, Germany
7. Deutsches Rheuma-Forschungszentrum (DRFZ), Berlin, 10117, Germany
8. Max Delbrück Center for Molecular Medicine in the Helmholtz Association, Berlin, 13125, Germany
9. Charité-Universitätsmedizin Berlin, Berlin, 13125, Germany
10. Berlin Institute of Health at Charité-Universitätsmedizin Berlin, Berlin, 10178, Germany

*Corresponding author: lynn-jeanette.savic@charite.de

Summary of Supporting Information Content

Table 1: Sequences of the primers for quantitative PCR.
Figure S1: Confirmation of polarization in M1-like and M2-like macrophages using real-time qPCR.
Figure S2: Effect of EU-VSOP and Synomag® on the proliferation and polarization of macrophages.
Figure S3: Effect of hyaluronidase and heparinase III on the viability of macrophages.
Figure S4: Co-localization of CD206-positive macrophages and iron-deposition from EU-VSOP in the peritumoral zone.
Details of methods: All the supplementary details of the method mentioned in the main text, including Supplementary Methods 1-7.

Table 1

Sequences of the primers for quantitative PCR.

Gene	Sequences	Gene	Sequences
CD206	F: TTCCTTTGGACGGATGGACG<br>R: TAAACTGCTTGAAGCGGCCA	Has 3	F：CTTAAGGGTTGCTTGCTTGC<br>R：GTTCGTGGGAGATGAAGGAA
IL -1 β	F：ATGATGGCTTATTACAGTGGCAA<br>R：GTCGGAGATTCGTAGCTGGA	CHSY1	F：AGTGTGTCTGGTCTTATGAGATGCA<br>R：AGCTGTGGAGCCTGTACTGGTAG
TNF- α	F：CCTCTCTCTAATCAGCCCTCTG<br>R：GAGGACCTGGGAGTAGATGAG	EXT 1	F：TCTTTACAGGCGGGAAGATG<br>R：GAAATCGAAGCAGGACTCCA
Has 1	F: CAAGATTCTTCAGTCTGGAC<br>R: TAAGAACGAGGAGAAAGCAG	GAPDH	F：GCACCGTCAAGGCTGAGAAC<br>R：TGGTGAAGACGCCAGTGGA

F: forward primer; **R**: reverse primer; ***CD206***: also known as the mannose receptor c-type 1 (MRC1), a marker for M2-like polarization; ***IL-1β***: Interleukin 1β, a marker for M1-like polarization; ***TNF-α***:Tumor necrosis factor α , a marker for M1-like polarization; ***Has*1**: Hyaluronan synthase 1,a key gene regulating the synthesis of hyaluronic acid; **Has3**: Hyaluronan synthase 3, a key gene regulating the synthesis of hyaluronic acid; ***EXT1***: Exostosin glycosyltransferase 1, a key gene regulating the synthesis of heparan sulfate; ***CHSY1***: Chondroitin sulfate synthase 1, a key gene regulating the synthesis of heparan sulfate.

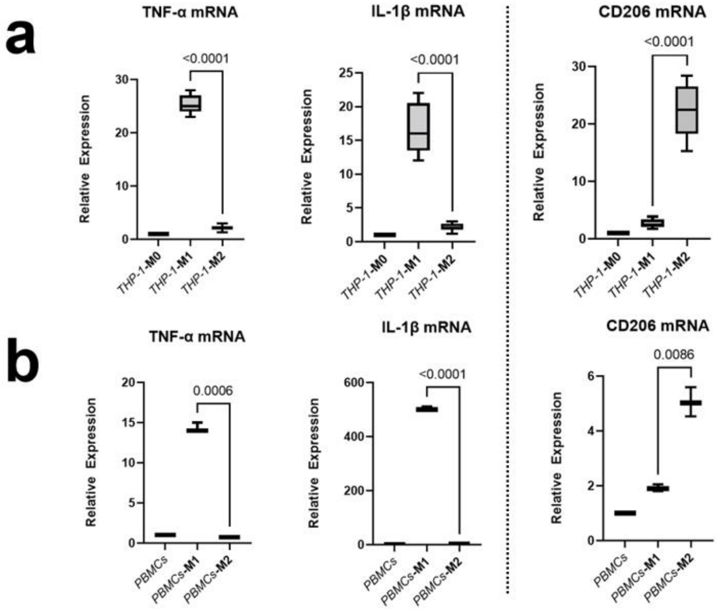

Supplementary Fig. 1 | Confirmation of polarization in M1-like and M2-like macrophages using real-time qPCR.

The whisker plots show increased expression levels of TNF-α and IL-1β in M1-like macrophages and increased CD206 expression in M2-like macrophages both originated from THP-1 cells (**a**)（n = 9) and PBMCs (**b**)（n = 3). Data were presented as mean ± SD. Two-tailed Student’s t-test was performed for (**a**) and (**b**).

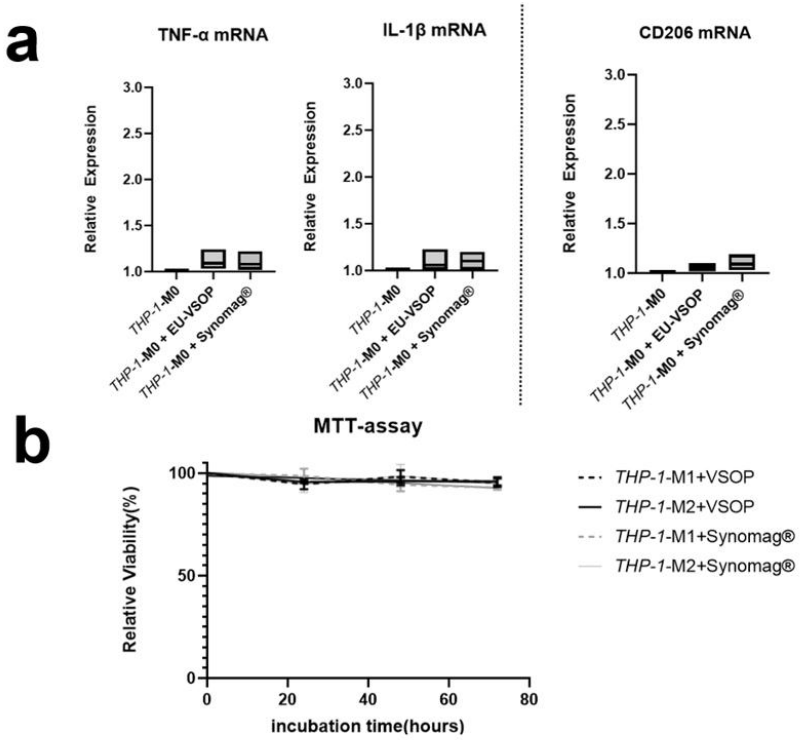

Supplementary Fig. 2 | Effect of EU-VSOP and Synomag® on the proliferation and polarization of macrophages.

EU-VSOP and Synomag® showed little impact on the mRNA levels of TNF-α and IL-1β (M1 markers) and CD206 (M2 marker) in unpolarized macrophages (**a**). Neither of the nanoparticles showed a significant impact on cell viability over a 72-hour period (**b**)（for **a** and **b**, *n* = 9). Data were presented as mean ± SD.

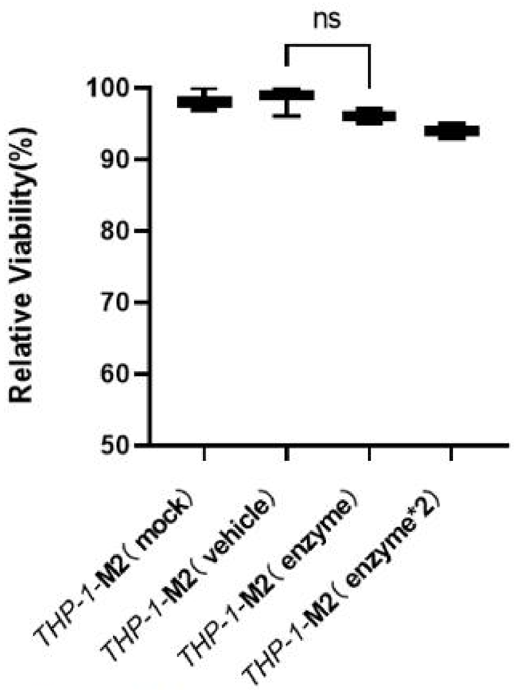

M2 (mock): M2 without treatment
M2 (vehicle): M2 treated with solvents
M2 (enzyme): M2 treated with hyaluronidase and heparinase III at working concentration
M2 (enzyme): M2 treated with hyaluronidase and heparinase III at double working concentrations

Supplementary Fig. 3 | Effect of hyaluronidase and heparinase III on the viability of macrophages.

Hyaluronidase and heparinase III demonstrated minimal impact on the viability of M2 polarized macrophages derived from THP-1 cells at the working concentration, compared to the vehicle groups (n = 3). Data are presented as mean ± SD.

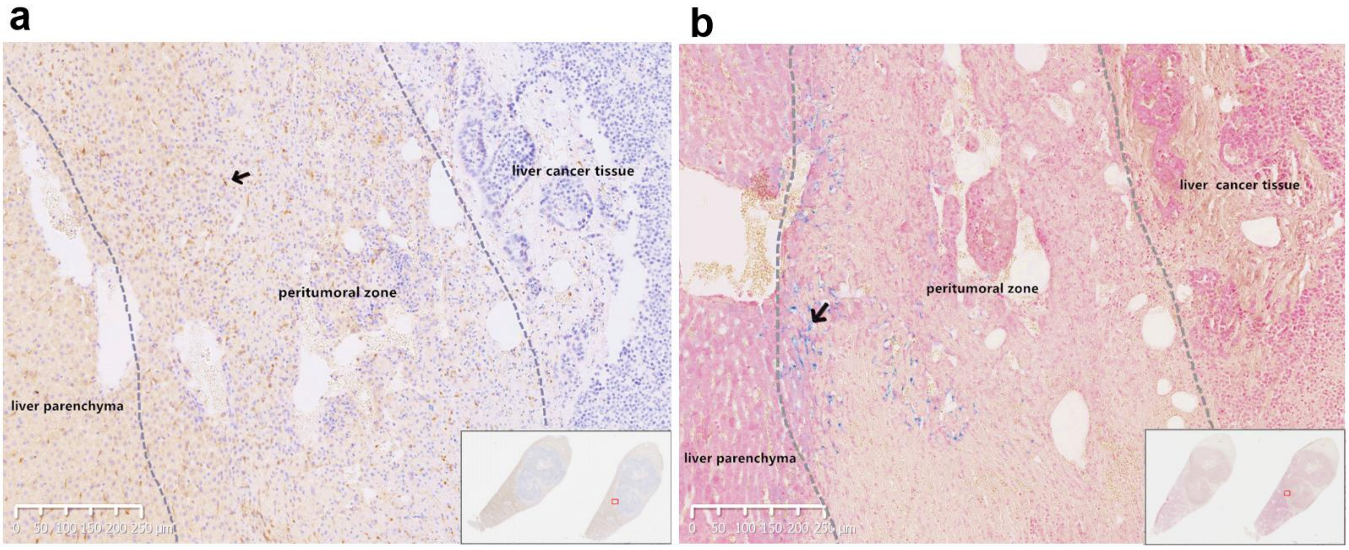

Supplementary Fig. 4 | Co-localization of CD206-positive macrophages and iron-deposition from EU-VSOP in the peritumoral zone.

Immunohistochemical staining (**a**) and Prussian blue staining (**b**) on the same pathological section of rabbit liver cancer tissues revealed significant partial overlap between CD206-positive areas and EU-VSOP deposition regions. The arrows point to the CD206 positive cells (**a**) and EU-VSOP detected by Prussian Blue staining (**b**).

Details of methods

Cell culture and induction of M0, M1-like, and M2-like macrophages polarization (Supplementary Methods 1)

THP-1 cells were purchased from the American Type Culture Collection (ATCC). The cells were cultured with 5% CO2 at 37°C in suspension in RPMI medium (1640; Invitrogen, Germany) containing 10% heat-inactivated fetal bovine serum (Gibco, #A5669801) and supplemented with 1% penicillin-streptomycin (Gibco, #15140122), which was herein defined as a complete medium. To isolate the primary PBMCs from a healthy donor, 30 mL whole blood was collected in EDTA tubes. The EDTA tubes were centrifuged (400 xg, 10 min, 4 ° C), the plasma was removed, and the cell pellet was resuspended with 1X BD Pharm Lyse Lysing Buffer (BD Biosciences, #555899) and incubated on ice for 15 mins. After centrifugation (400 xg, 10 min, 4 ° C) and aspiration of the supernatant, the pellet was resuspended with RPMI medium without FBS. The number of cells was quantified and then the cells were seeded in culture dishes or chamber slides in a cell incubator with 5% CO2 at 37 ° C. After 2 hours, the cells were gently washed twice with phosphate-buffered saline (PBS, Gibco, #C10010500BT) and incubated with complete RPMI medium for the subsequent experiment. One blood draw (20mL) yielded 3.8 million cells, so it was repeated multiple times for all experiments. The collection and utilization of human blood samples were conducted in accordance with the principles of the Declaration of Helsinki, under the supervision and approval of the ethics committee of Charité-Universitätsmedizin Berlin.

To induce M1-like and M2-like macrophages, THP-1 cells were differentiated into macrophages by 6 hours incubation with 150 PMA (Merck, #P1585-1MG), followed by a 24 h incubation in RPMI medium. M1-like polarization was induced by incubation with 20 ng/mL of IFN-γ (CST, #80385) and 100 ng/mL of LPS (Invitrogen, #00-4976-93). M2-like polarization was induced by incubation with 20 ng/mL of IL-4 (prospecbio, #CYT-211) and 20 ng/mL of IL-13 (prospecbio, #CYT-446). Adherent PBMCs were treated with granulocyte-macrophage colony-stimulating factor (GM-CSF) (bio-techne, #7954-GM-010/CF) and granulocyte colony-stimulating factor (G-CSF) (bio-techne, #214-CS-005/CFF) for 144 hours, respectively. The cells stimulated with GM-CSF were polarized to M1-like macrophages by incubation with 20 ng/mL of IFN-γ and 100 ng/mL of LPS. The cells treated with G-CSF were polarized to M2-like macrophages using 20 ng/mL of IL-4 and 20 ng/mL of IL-13.

Total RNA isolation and RT-qPCR (Supplementary Methods 2)

The total RNA of cells was extracted with TRIzol reagent (ABP Biosciences, #FP312A), and was quantified by Nanodrop 2000 spectrophotometer (Thermo Fisher). mRNA contained in 500 µg total RNA was reverse transcribed using random primer (Thermo, #48190-011), dNTP Mix (10 mM each, Thermo Fisher, #R0192), and SuperScript™ II Reverse Transcriptase kit (Invitrogen, #18064014). Amplification reaction assays contained power up SYBR Green Master Mix (Thermo Fisher, #A25776), and primers were synthesized by TIB Molbiol Syntheselabor GmbH (Berlin, Germany). mRNA abundance was quantified using the threshold cycle method in RQuantStudio™ 3 Real-Time PCR System (Applied Biosystems™, #A28572), according to the manufacturer's instructions.

GAG synthesis measurement with Click-iT® assays (Supplementary Methods 3)

M1-like and M2-like macrophages in 8-well adherent chamber slides (Falcon, #354118) were washed and incubated with RPMI medium containing Click-iT® GalNAz (tetraacetylated N-azidoacetylgalactosamine, Invitrogen, # C33365) and Click-iT® GlcNAz (tetraacetylated N-azidoacetylglucosamine, Invitrogen, #C33367) according to the manufacturer's instruction. After 48 hours, the cells were washed with PBS again and incubated with Alexa Fluor488 (Invitrogen, #C10405) for 1 hour, then washed by PBS twice gently. The cells were counterstained with Hoechst33342 (Thermo Fisher, #62249), washed again with PBS and sealed with Fluoromount™ mounting medium (Sigma, #F4680-25ML). Subsequently, the chamber slides were observed using an Axio Observer Z1 Zeiss microscope (Carl Zeiss microscopy GmbH, Germany), and the green fluorescence was analyzed by Image J (version 1.54 h; National Institutes of Health, USA) to compare the fluorescence intensity among different groups. The fluorescent signal intensities were measured with fluorescence levels captured within multiple Regions of Interest (ROIs) across specific areas of interest in the cells, and mean intensity values subsequently calculated for quantitative assessment.

Cell viability assay(Supplementary Methods 4)

Cell viability was assessed using the MTT assay. THP-1 cells were initially seeded in 96-well plates at a density of $1x10^4$ cells per well, followed by induction of M1 and M2-like macrophages as previously described. The medium was then replaced with RPMI medium supplemented with 1% FBS, and the macrophages were exposed to EU-VSOP and Synomag® at concentrations of 0.75 mM, respectively. At 0 h, 24 h, 48 h, and 72 h post-treatment, cell viability was evaluated using the MTT assay kit (abcam, #ab211091) according to the manufacturer's instructions. Absorbance readings were measured at 590 nm using a microplate reader. Cell viability data were presented as percentages relative to untreated controls.

Prussian blue staining of iron(Supplementary Methods 5)

Cells incubated in 8-well chamber slides were washed three times with PBS and fixed in 4% formalin solution for 20 minutes at RT. Formalin solution was removed and washed once with PBS. Prussian blue staining of iron was performed as previously described[16]. Briefly, cells were incubated with 1% potassium hexacyanoferrate (II) solution (Merk, #2440233) (5 minutes at RT) and 1% potassium hexacyanoferrate with 1% HCl (1 hour at RT), followed by washing three times with ddH2O, and counterstaining with nuclear red solution (Merck, #1001210500) for 1 minute. Slides were washed again for three times and dehydrated by dipping them into 70%, 80%, 96%, and 100% ethanol dilutions for 30 seconds each, followed by 5 minutes incubation in xylol and 5 minutes drying. Following a mounting gel covering, photomicrographs were obtained on an Axio Observer. Z1 Zeiss microscope and the iron density was quantified by Image J as described above.

Fluorescent microscopy of europium to quantify the uptake of EU-VSOP into macrophages (Supplementary Methods 6)

Cells were seeded into 8-well chamber slides with a density of $6 X 10^4$ cells/well and incubated with custom-made EU-VSOP[16] in medium with 1 % FBS at an iron concentration of 0.75 mM. After 48 hours, cells grown in chamber slides were washed three times with PBS prior to fixation with pre-cooled acetone-methanol (1:1) for 20 min. The fixation solution was removed and

samples were air-dried for 5 minutes. The cells were counterstained with 1ug/mL DAPI (Merck, #D9542-5MG) for 10 minutes at RT, then the DAPI solution was removed, and samples were washed twice with PBS. Pre-cooled DELFIA® Enhancer solutions (PerkinElmer, #C500-100) were added to the cells and incubated in the dark for 10 minutes at RT. The enhancer solutions were removed, and slides were air-dried for 5 minutes in the dark and mounted with coverslips and Fluoromount™ mounting medium as previously described[15]. The red fluorescence emitted by Europium was detected using a customized filter set consisting of an excitation filter (BP 350/50 nm), a beam splitter filter (380nm LP), and an emission filter (HC 615/20 nm) (AHF Analysentechnik AG, Germany). Photomicrographs were taken using the coordinates stored by the microscope software. Image J was used to compare red fluorescence intensity.

Magnetic particle spectroscopy (MPS) of the longitudinal ingestion of Synomag® in macrophages (Supplementary Methods 7)

MPS was utilized to measure the dynamic ingestion of Synomag®(micromod, #103-02-301) in macrophages. THP-1 cells were induced to polarized M1-like and M2-like macrophages and incubated with Synomag® for 0h, 2, 4, 8, 12, 24, 48, and 60 hours, and it is pertinent to specify that 0h designates cells untreated with Synomag®, thereby serving as the control group. Synomag® was prepared in medium with 1 % FBS and a final iron concentration of 0.75 mM (41 lg/mL). At each time point, cells were collected and washed three times with PBS to exclude the dead cells. Eventually, 10^6 cells were diluted in 30 μl PBS and assembled in a 0.2 mL PCR tube (Thermo Fisher, #4316567). After placing the tube into the MPS pick-up coil, repetitive measurements were started without Synomag® to check for magnetic impurities, before each sample was measured separately with MPS as previously described[17]. The mean ingested iron amount per cell could be estimated with the measured and reference measurement moment A3 (third harmonic value), and number of the cells (N) with the following formula: mean iron/cell (pg/cell)=($A3_{pellet}/A3_{ref}$) X($4.1X10^{-6}$/N).
